# Molecular Insights into Bacteriophage Evolution toward Its Host

**DOI:** 10.3390/v12101132

**Published:** 2020-10-06

**Authors:** Marina de Leeuw, Maayan Baron, Oshrit Ben David, Ariel Kushmaro

**Affiliations:** 1 Avram and Stella Goldstein-Goren Department of Biotechnology Engineering, Ben-Gurion University of the Negev, P.O. Box 653, Be’er Sheva 8410501, Israel; deleeuw@post.bgu.ac.il (M.d.L.); m2baron@health.ucsd.edu (M.B.); oshritbd@bgu.ac.il (O.B.D.); 2 The Novo Nordisk Foundation Center for Biosustainability, Technical University of Denmark, 2800 Kgs. Lyngby, Denmark; 3 The Ilse Katz Center for Meso and Nanoscale Science and Technology, Ben-Gurion University of the Negev, Be’er Sheva 8410501, Israel

**Keywords:** bacteriophage, bacteria, evolution, mutation, speciation, tubular protein B

## Abstract

Bacteriophages (phages), viruses that infect bacteria, are considered to be highly host-specific. To add to the knowledge about the evolution and development of bacteriophage speciation toward its host, we conducted a 21-day experiment with the broad host-range bacteriophage *Aquamicrobium* phage P14. We incubated the phage, which was previously isolated and enriched with the Alphaproteobacteria *Aquamicrobium* H14, with the Betaproteobacteria *Alcaligenaceae* H5. During the experiment, we observed an increase in the phage’s predation efficacy towards *Alcaligenaceae* H5. Furthermore, genome analysis and the comparison of the bacteriophage’s whole genome indicated that rather than being scattered evenly along the genome, mutations occur in specific regions. In total, 67% of the mutations with a frequency higher than 30% were located in genes that encode tail proteins, which are essential for host recognition and attachment. As control, we incubated the phage with the Alphaproteobacteria *Aquamicrobium* H8. In both experiments, most of the mutations appeared in the gene encoding the tail fiber protein. However, mutations in the gene encoding the tail tubular protein B were only observed when the phage was incubated with *Alcaligenaceae* H5. This highlights the phage’s tail as a key player in its adaptation to different hosts. We conclude that mutations in the phage’s genome were mainly located in tail-related regions. Further investigation is needed to fully characterize the adaptation mechanisms of the *Aquamicrobium* phage P14.

## 1. Introduction

Bacteriophages, or phages, are viruses that infect bacteria [1]. Consequently, phages have a marked influence on microbial populations [2,3,4]. Several studies in recent years have investigated the effects of phages on their host populations in natural environments [5,6], in the food industry [7,8,9,10,11], in agriculture [12], for medical research [13] and in wastewater treatment processes [14,15,16,17,18]. The high level of interest in phages is due to the potential of phage research not only to answer basic evolutionary questions, but also to lead to practical applications, e.g., fighting pathogens [7,8,19] and improving industrial processes [1,3,20].

Phage–host interaction is exceptional compared to other predator–prey associations. Phages are considered highly specific to their hosts, and their replication depends on the host cell’s machinery [21]. The first stage in the phage’s replication cycle is its attachment to the bacterium’s cell surface, during which a receptor on the tail or capsid of the phage recognizes receptor molecules on the host cell’s surface [22]. The ability of a phage to recognize and attach to those receptors is one of the factors that influences its host range [23]. After phage attachment to the bacterial host, it injects its genetic material into the cell. The subsequent stages in phage replication depend on the specific infection pathway [24].

To resist phage predation, bacteria exploit defense mechanisms that interfere with the phage adsorption or replication steps of the infection process [25]. Concurrently, the phage evolves novel mechanisms to enter the host’s cell and reproduce [26,27,28]. Both bacteria and phage undergo genetic changes that are favored by increased fitness and selection. The selection pressure that acts on one of the players (bacteria or phage) triggers a corresponding selection pressure on the other [29].

Previous studies have shown that mutations in viral genomes may help shift the parasite’s host range [30]. Ferris et al. [31] showed that random mutations in the host attachment gene of the RNA virus, bacteriophage φ6, enabled the phage to infect an additional strain of *Pseudomonas syringae*. Similarly, substitutions in the major capsid gene of the bacteriophage phiX174 were responsible for the phage’s adaptation to *Salmonella* and its decreased ability to infect *Escherichia coli* [32]. These experiments clearly demonstrated the abilities of phages to adapt to new hosts that are members of the same family as their original host.

In this study, we examined the ability of *Aquamicrobium* phage P14—isolated previously from a full-scale membrane bioreactor used to treat chemical industry wastewater, and propagated using *Aquamicrobium* H14—to adapt to the bacterium *Alcaligenaceae* H5 (isolated from the same reactor). Changes in the efficacy of phage predation toward *Alcaligenaceae* H5 were measured during a 21-day experiment, in which *Aquamicrobium* H14 served as the reference host. In addition, we incubated the phage with *Aquamicrobium* H8 (related to *Aquamicrobium* H14 and isolated from the same reactor). Samples from both experiments at different time points were sequenced and changes in the phage genome were monitored. The results demonstrate that rather than being evenly distributed across the phage genome, the mutations tend to cluster in tail-related regions, thus leading to increased phage predation efficacy toward *Alcaligenaceae* H5.

## 2. Materials and Methods

### 2.1. System Description

Both the phage and the bacteria samples were obtained previously from a full-scale membrane bioreactor used to treat chemical industry wastewater [15]. The phage, *Aquamicrobium* phage P14 (KX660669), is a broad host-range phage that was previously isolated using the bacteria *Aquamicrobium* H14 (GQ254284) as host. In contrast to most phages, *Aquamicrobium* phage P14 has the unusual ability to infect bacteria from different classes [33]. The phage is able to form plaques when plated with Alphaproteobacteria (*Aquamicrobium* H8 (GQ254278) and *Aquamicrobium* H14) and Betaproteobacteria (*Alcaligenaceae* H5 (GQ254275), *Alcaligenaceae* H13 (GQ254283) and *Alcaligenaceae* H17 (GQ254287)).

To examine the development of host specificity, we designed a phage-bacteria experiment. For this experiment we used *Aquamicrobium* phage P14 and two host bacteria: *Alcaligenaceae* H5 and, as the control for comparing the mutations in the genome, *Aquamicrobium* H8, which belongs to the same family as *Aquamicrobium* H14.

#### 2.1.1. Preparation

*Aquamicrobium* phage P14 was incubated with *Aquamicrobium* H14 for two days at 30 °C and constant shaking using 10 mL Luria–Bertani (LB) broth. The mixture of *Aquamicrobium* phage P14 with *Aquamicrobium* H14 was then filtered through a 0.22 μm Durapore PVDF membrane (Durapore^®^ PVDF membrane, Merck Millipore, Billerica, MA, USA). In parallel, *Alcaligenaceae* H5 and *Aquamicrobium* H8 were incubated in LB broth for two days in separate test tubes (30 °C, constant shaking).

#### 2.1.2. The Experiment

**Figure 1:**
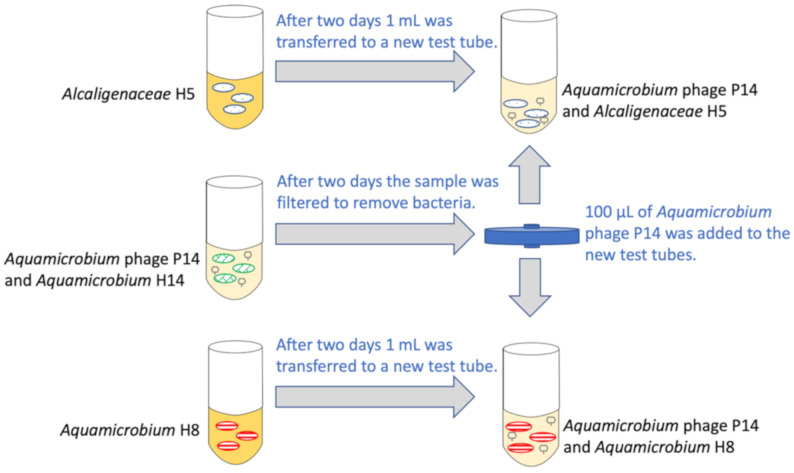
Schematic representation of the experimental setting.

At the beginning of the experiment, 1 mL of each bacteria sample was added to 10 mL of fresh LB broth (bacteria concentration: 2.33 × 10^7^ ± 0.39 × 10^7^ CFU/mL for *Alcaligenaceae* H5 and 2.73 × 10^6^ ± 1.63 × 10^6^ CFU/mL for *Aquamicrobium* H8). Then, 100 μL of *Aquamicrobium* phage P14 (7.72 × 10^8^ PFU/mL, counted with *Aquamicrobium* H14) was added to each test tube that contained the *Alcaligenaceae* H5 or *Aquamicrobium* H8 bacteria. See Figure 1 for a schematic presentation of the experimental setup.

The experiment was conducted once and continued for 21 days under batch conditions (30 °C, constant shaking), during which the medium was refreshed every 3–4 days by transferring 1 mL from each test tube to a new test tube containing 10 mL fresh medium. Approximately every six days, live counts (colony forming units, CFUs) were used to assess the bacterial concentrations, while plaque forming unit (PFU) assays were performed using the top agar procedure to enumerate the phages. Throughout the experiment, 16S rRNA gene sequencing was used to verify bacterial identity and to screen for contamination.

### 2.2. Sampling

Samples containing both bacteria and phage were taken every three to four days and stored at −80 °C in 20% glycerol for future analysis. Phage samples were obtained by centrifuging the biomass at 10,000× *g* for 5 min. The supernatant was filtered through a 0.22 μm Durapore PVDF membrane (Durapore^®^ PVDF membrane, Merck Millipore, Billerica, MA, USA) and kept frozen (−80 °C) until analysis (DNA extraction and sequencing).

### 2.3. Quantification of Bacteriophage and Bacteria

Viable bacterial counts were determined on days 0, 6, 13 and 20 by plating 20 μL of serial 10-fold diluted triplicate samples on LB agar plates. The plates were incubated at 30 °C for 48 h until the formation of colonies. Phages were quantified by counting PFUs on soft agar. Briefly, 100 μL quantities of serial 10-fold diluted phage samples were mixed with either *Aquamicrobium* H14 or *Alcaligenaceae* H5 in soft LB agar (0.7% agar in LB medium, 45 °C). The soft agar was then plated on warm LB agar plates (30 °C) and the plates were incubated for 48 h at 30 °C until plaques formed. Plaque counts and morphologies were documented.

### 2.4. DNA Extraction and Sequencing

Samples (1.5 mL) from the test tubes were filtered through a 0.22 μm syringe filter (Durapore^®^ PVDF membrane, Merck Millipore, Billerica, MA, USA) and then concentrated 10-fold using Amicon^®^ Ultra-0.5 3K Centrifugal Filters (Merck KGaA, Darmstadt, Germany). The DNA was extracted from the concentrated 0.15 mL samples using the UltraClean Microbial DNA Isolation Kit by MO BIO (Carlsbad, CA, USA) following the manufacturer’s protocol with two modifications: the first step of the DNA extraction, which concentrates bacterial cells by centrifugation, was omitted, and only 25 µL of elution solution was used. The purified DNA was sequenced on a MiSeq Illumina machine (San Diego, CA, USA) with 150 bp paired-end reads. The raw sequences used in this research are available in the NCBI’s Sequence Read Archive (SRA) under accessions SRX827032 and SRX7412192.

### 2.5. DNA Sequence Analysis, Bioinformatics and Comparative Analysis

Sequence analysis was performed using the CLC Genomics Workbench 12.0. The raw paired-end sequence reads were trimmed (ambiguous limit of 2, quality score limit of 0.05), and reads shorter than 15 nucleotides were discarded. To locate variants, reads from each sample were mapped to a reference genome previously assembled for *Aquamicrobium* phage P14 [33], GenBank database [34] accession KX660669. The mapping was performed using the default parameters (length fraction 0.5, and similarity fraction 0.8) and variants were detected using the quality variant detection function, in which only those variants with frequencies higher than 10% were annotated. Only SNPs with a forward/reverse balance greater than 0.05 and an average base quality of more than 20 were used, as was done previously [35]. The gene sequences with mutations were translated into protein sequences using the translate tool ExPASy (SIB Swiss Institute of Bioinformatics) [36].

## 3. Results and Discussion

*Aquamicrobium* phage P14 is a phage belonging to the *Autographiviridae* family [33]. It has icosahedral symmetry, it measures approximately 50 nm in length, it has a short tail, and its genome is 40,551 bp long. The phage used in this study was previously isolated from an industrial wastewater treatment plant [15] and enriched using *Aquamicrobium* H14 as the host. In contrast to most phages, however, it can infect bacteria from different classes. We therefore decided to explore this ability more rigorously by incubating the *Aquamicrobium* phage P14 with *Alcaligenaceae* H5 (which belongs to a different class than *Aquamicrobium* H14) and, as a control for comparing the mutations in the genome, with *Aquamicrobium* H8 (which belongs to the same family as the bacteria that was used during the phage enrichment stage). Since the experiment was performed once, more biological replicates are needed in order to strengthen our conclusions.

### 3.1. Changes in Bacterial and Bacteriophage Concentrations over Time

During the experiment, CFUs and PFUs were used to assess the bacterial concentration and to enumerate the phages (Table 1). Interestingly, bacterial concentrations did not decrease significantly during the experiment. This indicates that the bacteria gained some resistance to the phages, probably as a result of coevolution occurring along the experiment [27,37]. Another possible explanation for the relatively stable bacterial concentrations could be pseudolysogeny [38,39,40]. However, no significant morphological changes were observed in the bacterial colonies.

Curiously, the phage population remained marginally stable during the first two weeks of incubation with *Alcaligenaceae* H5. While refreshing the medium every several days could lead to a decrease in the phage concentration, the concentration calculated using *Alcaligenaceae* H5 for the plaque assay remained stable until day 13, indicating that the phages were able to propagate. Only on day 20 did the phage concentration decline; possible explanations for this may include phage inability to infect the host, in combination with natural decay and the dilution caused by the refreshment of the medium.

### 3.2. Changes in Phage Efficacy

**Figure 2:**
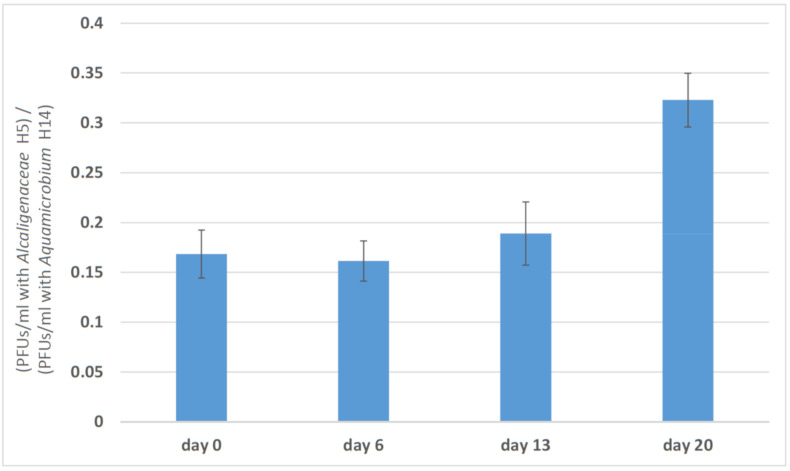
Changes in phage efficacy during the course of the experiment. Each column represents the ratio, at different time points, between the PFUs/mL of *Aquamicrobium* phage P14 when enumerated with *Alcaligenaceae* H5 (three technical replicates) and when enumerated with the *Aquamicrobium* H14 (three technical replicates). The results are shown as the mean ± standard deviation.

Phage predation efficacy toward *Alcaligenaceae* H5, defined by the ratio of the PFUs per ml obtained for the *Alcaligenaceae* H5 to the PFU measurement obtained with the original host *Aquamicrobium* H14, increased substantially on day 20 (Figure 2). The ability of a phage to increase its efficacy toward one of its hosts following incubation with it was demonstrated earlier for φX174 [32] and for bacteriophage T7 [41]. We did not determine whether the change in the infection ratio is due to an increase in phage predation efficacy toward *Alcaligenaceae* H5 or to a decrease in that efficacy toward *Aquamicrobium* H14. It should be noted, however, that even after 20 days, the phage PFU concentration enumerated by plating with *Aquamicrobium* H14 was higher than that found when the phage was plated with *Alcaligenaceae* H5.

### 3.3. Mapping Reads to the Genome of Aquamicrobium Phage P14

Reads from each sample were trimmed and then mapped to the reference genome of *Aquamicrobium* phage P14, which is 40,551 bp long. Most of the samples were successfully mapped. For the *Aquamicrobium* phage P14 that was incubated with *Alcaligenaceae* H5, 637,884 reads were mapped on day 3, 174,701 reads were mapped on day 9, 230,898 reads were mapped on day 15 and 164,871 reads were mapped on day 21. For the *Aquamicrobium* phage P14 that was incubated with *Aquamicrobium* H8, 89,965 reads were mapped on day 3, 155,520 reads were mapped on day 9, 201,079 reads were mapped on day 15, and 1,513,299 reads were mapped on day 21. Unfortunately, the sample at the beginning of the experiment contained a short DNA sequence which belongs to *Aquamicrobium* H14 (accession: MW015988). Therefore, only 1183 out of more than 3.5 million reads were successfully mapped (~0.03% of the reads) to the *Aquamicrobium* phage P14. Thus, we will focus mainly on mutations which occurred after day 3, mutations that became dominant along the experiment and mutations that were detected when the phage was incubated with one bacteria but not with the other.

### 3.4. Mutations in the Phage Genome

**Figure 3:**
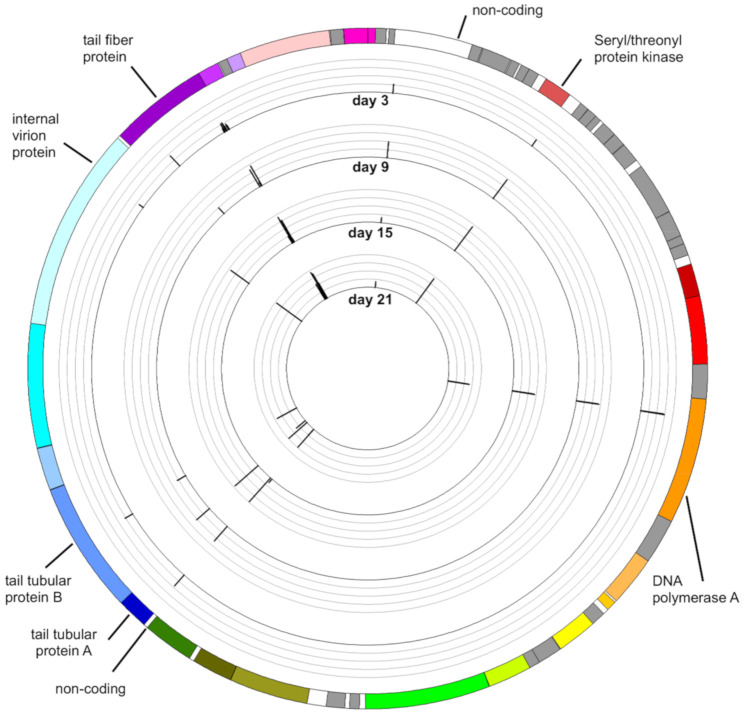
Variance from the reference genome of *Aquamicrobium* phage P14 over the course of the experiment. By each position at which the frequency of a mutation was higher than 10% of the mapped reads, a bar is shown to indicate the frequency (0–100%, grey line every 25%). Genes encoding hypothetical proteins are marked in grey while genes encoding proteins with an identified function are marked in color. The image was produced using Circos [42].

The distribution of variants along the genome of *Aquamicrobium* phage P14 during incubation with *Alcaligenaceae* H5 is given in Figure 3, in which the variance frequency threshold was 10% (i.e., a minimum of 10% of the reads mapped to a specific spot had a mutation at that spot). When using a 30% variance frequency threshold, a total of 18 mutations were detected along the phage genome during the experiment (Table 2). From among these mutations, one was only detected on day 3, two were only detected on day 9 and five were only detected on day 21. All other 10 mutations were present in at least two time points.

#### 3.4.1. Mutation in the Gene Encoding Seryl-Threonyl Protein Kinase

One notable mutation was seen in the gene encoding seryl-threonyl protein kinase, in which two nucleotides were deleted at positions 4099 and 4100. These deletions interfered with the stop codon, and instead of a glutamic acid at the end of the protein’s sequence, 20 amino acids were added to the C-terminus: VTCSINVRAWSCFGCRTGRV. It is not clear, however, what influence, if any, this addition has. A few matches to the new C-terminus were found using blastp (protein to protein) [43]. None of them were significant, however, since the sequence is relatively short.

The mutation was observed when the *Aquamicrobium* phage P14 was incubated with *Alcaligenaceae* H5, on days 9, 15 and 21 (see Table 2). It was also observed when the phage was incubated with *Aquamicrobium* H8 (on days 3 and 9), but in this case, the frequency of the mutation was significantly lower, and on day 21 it was found in only 13.15% of the mapped reads (see Table S2). It is possible that this mutation occurred during the preparation of the experiment (from the beginning of the experiment, only four reads were mapped to this location, and one of them had the same mutation). Interestingly, the frequency of this mutation increased only during incubation with *Alcaligenaceae* H5, although this could be a coincidence and further investigation is needed.

#### 3.4.2. Mutation in the Gene Encoding DNA Polymerase A

Two mutations were annotated in the gene encoding DNA polymerase. In both cases, a guanine was replaced by thymine (locations 11,077 and 11,086). As a result, glycine_134_ and glycine_137_, which have non-polar side chains, became valines, which have a hydrophobic side chain. Although such a switch may have marked effects on protein structure and function, we do not have enough data to support such a claim. Unfortunately, only two reads from the sample at the beginning of the experiment were mapped to this area, and both of them had thymine in locations 11,077 and 11,086. In addition, in both the experimental (incubation with *Alcaligenaceae* H5) and control (incubation with *Aquamicrobium* H8) samples, about two-thirds of the mapped reads had this mutation, and no significant increase or decrease was observed in the mutation’s frequency over the course of the experiment.

**Figure 4:**
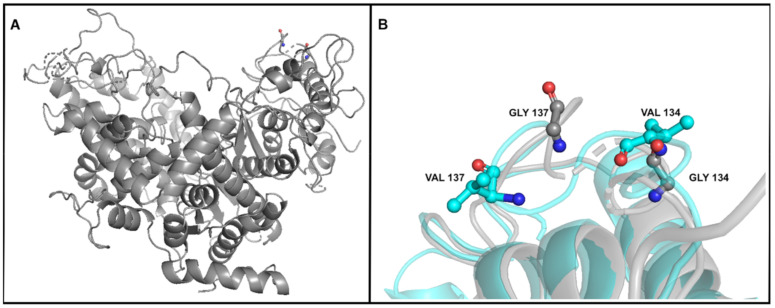
The predicted structure of the DNA polymerase A constructed using Phyre2 [44]. (**A**) The whole structure of the protein; Glycines 134 and 137 can be seen in the upper right corner. (**B**) Focus on amino acids 134 and 137. Grey: the structure with Glycines; Cyan: the structure with Valines.

Notably, these two mutations tend to appear together. For example, in 87% of the reads in which thymine is in position 11,077, a thymine is also in location 11,086, and 92% of the reads with a thymine in location 11,086 (same sample) also have a thymine in location 11,077. We used Phyre2 to predict the 3D structure based on the known structures of DNA polymerase proteins [44]. As can be seen in Figure 4, the affected amino acids are glycine_134_ and glycine_137_, both of which are replaced by a valine. According to the 3D structure prediction, these amino acids are located in a segment of a loop in which the distance between the alpha carbons of the glycines is 7.3 Å.

#### 3.4.3. Mutation in the Genes Encoding the Tail Tubular Proteins

One mutation in location 24,958 was marked in the gene encoding the tail tubular protein A. In this case, a cytosine was replaced by a thymine. This mutation caused the replacement of a proline_21_, an amino acid with a non-polar side chain, with a leucine, which has a hydrophobic side chain. Interestingly, the frequency of this mutation changed along the timeline of the experiment. When the *Aquamicrobium* phage P14 was incubated with *Alcaligenaceae* H5 it increased from 44.52% on day 3 to 59.76% on day 9, and 98.11% on day 15. Then, it decreased on day 21 to 73.25%. However, during phage incubation with *Aquamicrobium* H8, this mutation was also observed (on days 3 (12.45%), 9 (34.76%) and 15 (79.93%)). Therefore, it is possible that the mutation already occurred during the preparation of the experiment.

**Figure 5:**
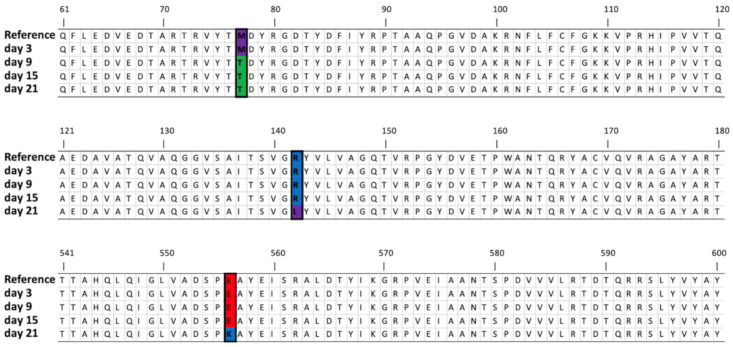
Amino acid sequences (61–180 and 541–600) of the tail tubular protein B and the mutations that appeared during the experiment (colored by polarity). Note: for simplicity, all mutations that occurred with more than 30% frequency at a certain time point are shown on the same protein sequence; this does not necessarily mean, however, that all the mutations occurred on the same specific protein sequence.

Five mutations were noted during the experiment in the gene encoding the tail tubular protein B. One of them first appeared on day 9 and remained throughout the experiment (location 25,728: thymine replaced by cytosine). Each of the other four mutations was only observed once; on day 3, a mutation was annotated on location 26,835 (27.03% of mapped reads), on day 9 on location 26,976 (29.73% of mapped reads) and on day 21 mutations were annotated on locations 25,923 (35.97% of mapped reads) and 27,164 (67.64% of mapped reads). It is possible that some of these mutations were responsible for the observed changes in phage predation efficacy toward *Alcaligenaceae* H5 over the course of the experiment. The influence of the mutations (those with frequencies higher than 30%) on the protein sequence is shown in Figure 5. Remarkably, no mutations were seen in this gene when the phage was incubated with *Aquamicrobium* H8.

Tubular proteins A and B form a tubular structure surrounded by tail fibers [45]. Tubular protein A is responsible for the attachment of the fibers, while tail tubular B forms the end of the tail [45]. In addition to their structural function, recent studies suggest that the tail tubular proteins have a role in host recognition and attachment to its surface [46,47]. Furthermore, the tail tubular protein A of the *Klebsiella pneumoniae* phage KP32 was found to exhibit lytic activity towards exopolysaccharide [48]. These studies highlight the possibility that the tail tubular proteins of *Aquamicrobium* phage P14 have multiple functions, and that changes in their amino acids can influence not only the injection of viral DNA into the host, but also the attachment to the host’s receptors.

#### 3.4.4. Mutations in Genes Encoding Internal Virion Proteins

Two mutations were observed in genes encoding internal virion proteins. In one mutation, marked at position 34,414, a thymine was replaced by guanine on day 3 in 15.65% of the reads. By day 21, this mutation became dominant and 95.04% of the mapped reads had a guanine in this location. This dominance suggests that the mutation gave the phage some advantage over phages that lacked it. While mutations in the internal virion protein may seem irrelevant to phage predation abilities, research conducted on bacteriophage T7 demonstrated the opposite. In the case of bacteriophage T7, the internal virion proteins are believed to play a major role in the ejection of the viral genome from the phage capsid into the bacterial cell [49]. In addition, mutations in these proteins were shown to have important implications for phage predation abilities [50].

Since only three reads were mapped to this location at the beginning of the experiment (none of them had the mutation), the possibility that this mutation occurred during phage preparation cannot be dismissed. Nonetheless, this mutation was not detected in any of the samples when the phage was incubated with *Aquamicrobium* H8. Thus, even if it occurred during phage preparation, it only became dominant when the phage was incubated with *Alcaligenaceae* H5.

A mutation in another internal virion protein (location 30,484) appeared only when the phage was incubated with *Aquamicrobium* H8. In this case, the frequency of the mutation did not increase over the course of the experiment, peaking with a frequency of 34.96% of the reads on day 3, then falling to 10.40% of the reads on day 9 and 12.88% of the reads on day 21.

#### 3.4.5. Mutations in the Gene Encoding the Tail Fiber Protein

In total, 20 mutations were annotated in the gene encoding the tail fiber protein when *Aquamicrobium* phage P14 was incubated with *Alcaligenaceae* H5 (10% frequency threshold, Table S1). Out of them, only nine were observed in more than 30% of the mapped reads and will be discussed further.

One mutation, which was only present on day 3 (in location 35,714) and when the phage was incubated with *Aquamicrobium* H8 (the control), was probably introduced during the experiment preparation. Another mutation (in location 37,165) was present 9 days after the beginning of the experiment, but was not detected in the following samples. Three mutations emerged on day 15 and were still present at high frequencies on day 21, and another three emerged only on day 21 (see Table 2).

Remarkably, the nucleotide in location 37,092 (originally thymine) was replaced in the majority of mapped reads by guanine on day 9, by adenine on day 15 and again by guanine on day 21. Each mutation resulted in a different amino acid in location 609 of the phage’s tail fiber protein. However, in both cases the Isoleucine (an amino acid with a hydrophobic side chain) was replaced by an amino acid with a positively charged side chain. On days 9 and 21, the Isoleucine was replaced by Arginine, while on day 15 it was replaced by Lysine. It is possible that a positively charged side chain in this location enhances the phage’s ability to attach to *Alcaligenaceae* H5. Nevertheless, further investigation is needed in order to confirm this hypothesis.

**Figure 6:**
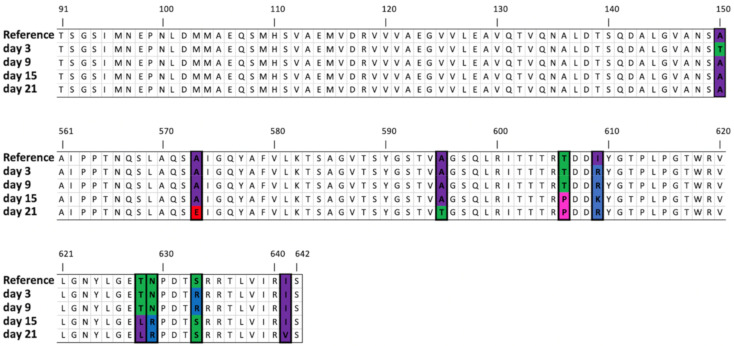
Amino acid sequence (91–150 and 561–642) of the tail fiber protein showing the mutations that appeared during the experiment (colored by polarity). Note: for simplicity, all mutations that occurred with more than 30% frequency at a certain time point are shown on the same protein sequence; this does not necessarily mean, however, that all the mutations occurred on the same specific protein sequence.

Figure 6 shows an alignment of the translated protein sequences. All nine mutations were nonsynonymous, and eight of them influenced the translated amino acid sequence near the C-terminus. This end of the protein sequence does not have any blast [51] alignment (hits). In contrast, the N-terminus has a blastx [43] match (E-value: 1.28 × 10^−11^) to the phage T7 tail fiber protein superfamily (pfam03906). This is the point at which the tail fiber protein of the bacteriophage T7 attaches to the phage’s tail, while the C-terminus is the side that recognizes and attaches to the host [52].

This observation correlates well with the findings of previous studies with RNA and DNA viruses [53,54,55,56]. Tétart et al. (1996) and Le et al. (2013) demonstrated that changes in the C-terminus of the tail fiber protein lead to changes in the host range of the phage [55,56]. Thus, mutations in the C-terminus of its tail fiber protein confer on the phage the ability to attach to and infect new hosts, while the N-terminus, which attaches to the phage’s tail, is conserved.

#### 3.4.6. Mutations in Non-Coding Sequences

When using the 10% frequency threshold, two mutations in non-coding regions are evident (Figure 3). The mutation in location 24,876 appeared only when *Aquamicrobium* phage P14 was incubated with *Alcaligenaceae* H5 and only on day 15 (13.87% of the mapped reads). The other mutation was the insertion of a cytosine between nucleotides 570 and 571. This mutation appeared both when the phage was incubated with *Alcaligenaceae* H5 and when incubated with *Aquamicrobium* H8. Therefore, it is possible that it already occurred during the preparation of the experiment. Since the frequency of these mutations did not increase over time, it can be assumed that they did not confer on the phage any advantage.

#### 3.4.7. Distribution of Variants along the Genome

Mutations were mainly observed in specific genes and were not evenly spread over the entire genome. Among the 15 mutations identified on day 21 (using a 30% frequency threshold), 7 were located in the gene encoding the tail fiber protein (46.7% of the mutations), which is only 1929 bp long (4.76% of the genome). Furthermore, one mutation was located in the gene encoding the tubular tail protein A, and three were identified in the gene encoding tubular tail protein B. Thus, 11 out of 15 mutations (73.3%) were located in tail-associated genes (Table 2).

To explore the tendencies of specific genes to mutate, the number of mutations observed in a gene (in the whole phage community at a certain time point) was divided by the gene’s length. The average variances from the reference genome were 0.10, 0.20, 0.25 and 0.37 mutations per 1000 bp for days 3, 9, 15 and 21, respectively. The average variances for the tail fiber protein were 0.5, 1.0, 2.1 and 3.6 mutations per 1000 bp for days 3, 9, 15 and 21, respectively. Thus, the average variance for the gene encoding the tail fiber protein was significantly higher than that for the whole genome.

There are several possible explanations for this observation, and they raise important evolutionary questions. First, it is possible that while mutations are created randomly along the genome, some mutations, those that confer on the phage an advantage, become frequent. Thus, selection stress in the environment may dictate which genes show a tendency for mutation. On the other hand, it is also possible that the genes that enable the phage to shift its host range are more prone to mutation, and are not as sensitive to modification as other genes. These questions are also relevant to specific regions within a single gene, such as that of the gene encoding the tail fiber protein. In the case of this gene, the mutations were mainly located in a region which encodes the area near the C-terminus of the tail fiber protein; an area which is used in host recognition.

The fact that most of the observed mutations occurred in tail-related genes correlates well with previously published work by Yosef et al. (2017) [57]. In their work, the researchers extended the host range of a T7 phage by designing hybrid particles that displayed various phage tail and tail fiber proteins. Similarly, the random mutations in the tail-related genes of *Aquamicrobium* phage P14 allow it to explore new infection strategies that are suited to *Alcaligenaceae* H5.

Interestingly, in the study by Crill et al. (2000) [32], φX174 showed mutations in almost all of its genes as a result of switching hosts. However, more than half of the reversions (nucleotides that swapped back to the previous nucleotide due to incubation with the previous host) were spotted in the gene encoding the major capsid protein. Since φX174 has no tail, the major capsid protein is responsible for phage interaction with the host cell membrane and receptors. Taken together, these findings and observations strengthen the conclusion that the phage’s ability to rapidly alter the proteins responsible for host recognition allows it to adapt to new hosts.

## 4. Conclusions

In this study, the *Aquamicrobium* phage P14, a phage belonging to the *Autographiviridae* family, was grown with *Alcaligenaceae* H5 for 21 days. This experiment allowed us to monitor the mutations that occurred in the phage genome over time, and to observe changes in phage efficacy toward its host. Indeed, during the 21-day experiment, the phage efficacy toward the *Alcaligenaceae* H5 host increased. Furthermore, mutations in the genome were observed in specific regions, and mostly in the gene encoding the tail fiber protein, which is responsible for host recognition. The experiment was performed once and therefore more biological replicates are needed in order to confirm our conclusions. In addition, further investigation is needed to answer basic evolutionary questions about the tendency of specific genes to mutate, and the implications of those mutations for host shifts and speciation.

## Figures and Tables

**Table 1:** *Alcaligenaceae* H5 and *Aquamicrobium* phage P14 concentrations during the experiment (phage concentration was determined using *Alcaligenaceae* H5 for the plaque assay).

Day	Bacteria Concentration (CFU/mL)	Phage Concentration (PFU/mL)
0	2.3 × 10^7^ ± 3.9 × 10^6^	1.3 × 10^6^ ± 6.5 × 10^5^
6	1.2 × 10^7^ ± 2.0 × 10^6^	1.9 × 10^7^ ± 1.9 × 10^6^
13	9.7 × 10^6^ ± 1.6 × 10^6^	3.1 × 10^7^ ± 1.4 × 10^6^
20	2.9 × 10^7^ ± 1.3 × 10^6^	1.3 × 10^6^ ± 9.8 × 10^5^

**Table 2:** Variants (with frequency above 30%) in the *Aquamicrobium* phage P14 genome over time when incubated with *Alcaligenaceae* H5 and when incubated with *Aquamicrobium* H8. Frequency is indicated in parentheses. Variant types: SNV—single nucleotide variant; MNV—multiple nucleotide variant; insertion—addition of a nucleotide; and deletion—removal of a nucleotide. t *—the nucleotides in mapped reads present at the beginning of the experiment; due to the low coverage, the number of mapped reads is given in parentheses.

					Incubation with *Alcaligenaceae* H5				Incubation with *Aquamicrobium* H8				
Gene	Position	Type	Reference	t * = 0	Day 3	Day 9	Day 15	Day 21	Day 3	Day 9	Day 15	Day 21	Changes in Protein Sequence
-	570^571	Insertion	-	C(3), (1)		C (50.27%)				C (32.76%)	C (42.70%)		-
Seryl-threonyl protein kinase	4099..4100	Deletion	AG	A(3), (1)		(71.11%)	(91.83%)	(93.37%)	(30.55%)	(31.69%)			Glu182-> VTCSINVRAWSCFGCRTGRV
DNA polymerase A	11,077	SNV	G	T (1)	T (70.58%)	T (65.97%)	T (63.01%)	T (64.44%)	T (69.88%)	T (63.34%)	T (70.53%)	T (70.23%)	Gly134->Val
	11,086	SNV	G	T (2)	T (73.98%)	T (69.46%)	T (66.45%)	T (66.07%)	T (69.64%)	T (63.40%)	T (72.76%)	T (72.84%)	Gly137->Val
Tail tubular protein A	24,958	SNV	C	C (2)	T (44.52%)	T (59.76%)	T (98.11%)	T (73.25%)		T (34.76%)	T (70.03%)		Pro21->Leu
Tail tubular protein B	25,728	SNV	T	-		C (52.85%)	C (93.92%)	C (74.19%)					Met77->Thr
	25,923	SNV	G	G (2)				T (35.97%)					Arg142->Leu
	27,164	SNV	G	G (1)				A (67.64%)					Glu556->Lys
Internal virion protein	30,484	SNV	C	C (6), T(2)					T (34.96%)				Leu534 ->Phe
Internal virion protein	34,414	SNV	T	T (3)			G (68.26%)	G (95.04%)					His1055->Gln
Tail fiber protein	35,714	SNV	G	G(3), A(1)	A (42.91%)				A (35.95%)	A (56.24%)	A (82.92%)		Ala150 -> Thr
	36,984	SNV	C	C (4)				A (54.63%)					Ala573 -> Glu
	37,023	SNV	G	G(2), T(1)						T (31.58%)	T (39.81%)		G586->Val
	37,049	SNV	G	C(1), G(1)				A (37.67%)			A (35.67%)		Ala595 -> Glu
	37,082	SNV	A	A (3)			C (79.15%)	C (59.96%)					Glu606-> Pro
	37,085	SNV	G	G (3)							A (37.98%)		D607->Asn
	37,092	SNV	T	T (3)		G (60.30%)	A (72.33%)	G (93.39%)					Days 9 and 21: Ile609->Arg Day 15: Ile609->Lys
	37,146..37,154	Deletion	AGACTAACC	AGA * TAACC (2)					(40.84%)	(37.48%)			ETNP->Ala627
	37,148..37,149	MNV	AC	AC (1), AT (1)			CT (76.17%)	CT (86.69%)					Thr628->Leu
	37,151..37,152	MNV	AA	AA (2)			CG (77.18%)	CG (87.24%)					Asn629->Arg
											AG (44.08%)		Asn629->Ser
	37,155	SNV	C	C (2)							A (30.27%)		Pro630->His
	37,165	MNV	T	T (3)		A (70.17%)			A (51.20%)				Ser633->Arg
	37,187	SNV	A	A (5)				G (53.73%)					Ile641->Val

## Supplementary Materials

The following are available online at https://www.mdpi.com/1999-4915/12/10/1132/s1, Table S1: Variants (with frequency above 10%) in the *Aquamicrobium* phage P14 genome over time when incubated with *Alcaligenaceae* H5, Table S2: Variants (with frequency above 10%) in the *Aquamicrobium* phage P14 genome over time when incubated with *Aquamicrobium* H8.
